# Nitroalkene reduction in deep eutectic solvents promoted by BH_3_NH_3_

**DOI:** 10.3762/bjoc.17.83

**Published:** 2021-05-06

**Authors:** Chiara Faverio, Monica Fiorenza Boselli, Patricia Camarero Gonzalez, Alessandra Puglisi, Maurizio Benaglia

**Affiliations:** 1 Dipartimento di Chimica, Università degli Studi di Milano, Via C. Golgi, 19, I-20133, Milano, Italy

**Keywords:** alternative solvents, atom economy, DES, nitro derivatives, reduction

## Abstract

Deep eutectic solvents (DESs) have gained attention as green and safe as well as economically and environmentally sustainable alternative to the traditional organic solvents. Here, we report the combination of an atom-economic, very convenient and inexpensive reagent, such as BH_3_NH_3_, with bio-based eutectic mixtures as biorenewable solvents in the synthesis of nitroalkanes, valuable precursors of amines. A variety of nitrostyrenes and alkyl-substituted nitroalkenes, including α- and β-substituted nitroolefins, were chemoselectively reduced to the nitroalkanes, with an atom economy-oriented, simple and convenient experimental procedure. A reliable and easily reproducible protocol to isolate the product without the use of any organic solvent was established, and the recyclability of the DES mixture was successfully investigated.

## Introduction

The search for alternative solvents, not derived from oil but from biorenewable resources, is a topic of primary importance in modern chemistry [1–2]. The solvents are the major contributor to the waste generated in chemical industries, and the elimination or replacement of these with more sustainable alternatives is part of the efforts of the whole research community concerned with the concept of a circular economy [3].

In this context, deep eutectic solvents (DESs) have attracted an increasing attention as green, safe, economically and environmentally sustainable alternative to the traditional organic solvents [4]. They are combinations of two or three naturally occurring components, able to engage in reciprocal hydrogen bond interactions to form an eutectic mixture with a melting point lower than that of either of the individual components. DESs feature several favorable properties: they do not need any purification and the physicochemical properties can be easily tuned and designed to meet specific requirements; they also offer the possibility to develop convenient methodologies to isolate the product by extraction or precipitation, and thus making the reuse of the DES mixture feasible. The large number of biodegradable raw materials that can be used to form eutectic mixtures offers an incredibly high variety of combinations to generate new, safe and biodegradable DESs [5].

Another fundamental principle of green chemistry is the atom economy concept. In this context, ammonia borane (AB) is receiving increasing attention as relatively inexpensive, useful reduction reagent for developing new green synthetic transformations [6–12]. Compared to other bioinspired reagents, such as Hantzsch esters, AB represents a more convenient reagent that generates much less waste [10]. We have recently reported the use of ammonia borane in the chemoselective reduction of a variety of nitrostyrenes and alkyl-substituted nitroalkenes to the corresponding nitroalkanes, without any catalyst or additive [13].

We thought that the combination of an atom economic, very convenient and inexpensive reagent such as BH_3_NH_3_ in bio-based eutectic mixtures as biorenewable alternative solvents in the synthesis of nitroalkanes, valuable precursors of amines, would represent a significant step towards the development of more sustainable synthetic organic methodologies for the preparation of valuable molecules. In the present communication, we report the results of our explorative studies, aimed to develop a chemoselective nitroalkene reduction in DESs, with the goals to establish a reliable and reproducible protocol to isolate the product without the use of any organic solvent and to assess the recovery and the recyclability of the DES mixture [14].

## Results and Discussion

Based on our previous experiences with organocatalytic reactions in alternative, biodegradable solvents [15–16], and following some preliminary investigations on the physicochemical properties of several DES combinations, for this application, we decided to focus our attention on the use of some choline chloride (ChCl)-based eutectic mixtures as reaction media.

The reduction of β-nitrostyrene to afford (2-nitroethyl)benzene was selected as model reaction, and it was performed typically in the presence of 1 molar equiv of ammonia borane for 18 h at 60 °C (see Table 1).

**Table 1:** Preliminary studies on AB-mediated nitrostyrene reduction in different solvent systems.

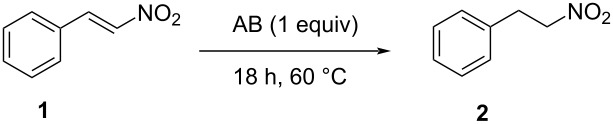				

entry	DES	components	substrate *c*, M	yield, %

1	DES A	ChCl/urea 1:2	0.5	7
2	DES B	ChCl/gly 1:2	0.5	27
3	DES C	ChCl/fructose/H_2_O 1:1:1	0.5	n.d.
4	glycerol (gly)	gly	0.5	41
5	water	water	0.5	<5
6	DES B	ChCl/gly 1:2	0.1	41
7^a^	DES B	ChCl/gly 1:2	0.1	30
8	gly	gly	0.1	61
9	water	water	0.1	7

^a^Reacton time 4 h.

First, the behavior of three eutectic mixtures was investigated in the reduction performed at a 0.5 M substrate concentration (entries 1–3, Table 1). A low yield of product **2** and a significant amount of unreacted starting material were observed for DESs A and C; only with a ChCl/gly mixture, DES B, the product was isolated in 27% yield. For sake of comparison, the reaction was also performed in water, where a negligible amount of the product was detected, and in glycerol, which proved to be a good reaction medium (41% yield) [17]. In order to further speed up the reaction and suppress the byproducts formation [18], the reaction was performed at 60 °C for 18 h on a 0,1 M substrate concentration (entries 6–9, Table 1).

Under those conditions, the product was isolated after chromatographic purification in a higher yield; in DES B, 41% yield was reached, while in glycerol, the reaction gave 61% yield (entries 6 and 8, Table 1). Therefore, DES mixture B (ChCl/gly 1:2) and glycerol were selected as reaction media of choice to further investigate the reduction of nitroalkenes mediated by ammonia borane. Both solvent systems represent promising alternatives, green solvents for organic reactions; glycerol is a nontoxic, biodegradable and nonflammable solvent for which no special handling or storage precautions are required [19]. Some limits, such as high viscosity and low solubility of highly hydrophobic compounds and possible side reactions due to the presence of hydroxy groups, can be overcome by adding a cosolvent; indeed, glycerol is often used as a component of eutectic mixtures, such as in DES B, as in this work.

In a general procedure, nitroolefin (0.4 mmol) and ammonia borane (0.4 mmol) were added to 4.5 g of freshly prepared DES. After the reaction mixture was heated at 60 °C for 18 h, the reaction was cooled to room temperature, and the product was isolated, either by adding 4 mL of water, which dissolves the DES, and extracting the nitroalkane with AcOEt, or by direct separation of the organic residue from the eutectic mixture (see below for recycling experiments and Supporting Information File 1 for details on the different work-up procedures).

**Scheme 1:**
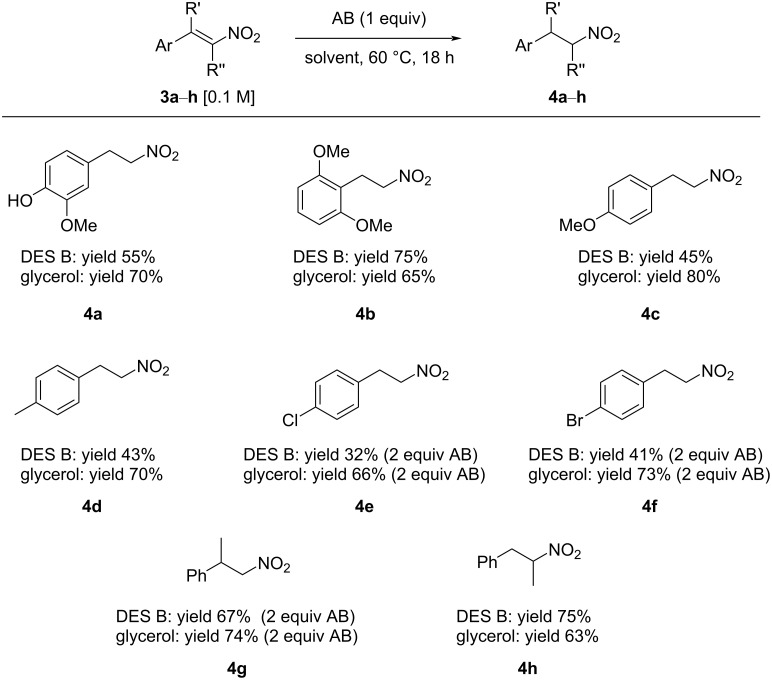
AB-mediated reductions of nitrostyrenes **3a**–**h**.

The synthetic applicability was then investigated by studying the reduction of differently substituted aryl- and alkylnitroalkenes. The reaction was successfully performed with different nitrostyrenes (Scheme 1). Electron-rich nitroalkenes were reduced in fair to very good yield (up to 80% yield, see products **4a**–**d** in Scheme 1). The reaction of substrates featuring unprotected hydroxy groups is possible (see product **4a**). The reaction of substrates bearing electron-withdrawing residues with ammonia borane was observed to proceed slower, and the use of two equiv of NH_3_BH_3_ was necessary to speed up the reaction and obtain the products with good yield after 18 hours (see adducts **4e**–**f**). For those less reactive compounds, glycerol proofed to be a better reaction solvent.

The reduction of either α- or β-substituted nitrostyrenes was successfully accomplished both in glycerol and in DES B, with the eutectic mixture generally performing better than glycerol alone; indeed, α-substituted nitroalkane **4h** was isolated in 75% yield after 18 h of reaction at 60 °C. We further explored other DESs, and in particular, we turned our attention to betaine-containing eutectic mixtures DES D and E, and a ChCl/ʟ-(+)-lactic acid mixture, DES F (Table 2).

**Table 2:** Preliminary studies on AB-mediated nitrostyrene reduction in DESs **D**–**F**.

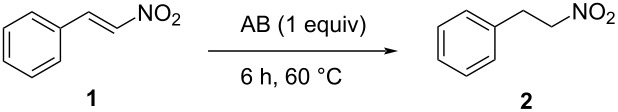				

entry	DES	components	substrate *c*, M	yield, %

1	DES D	betaine/glycolic acid 1:2	0.75 M	73
2	DES E	betaine/gly 1:2	0.75 M	n.d.
3	DES F	ChCl/ʟ-(+)-lactic acid 1:3^a^	0.75 M	30

^a^A 90% solution of lactic acid in water, dried on CaCl_2_ pellets, was used.

The reduction worked in all systems, in low yield with DES E (where it was not possible to separate the product from the DES) and DES F, but it proceeded in very high yield and short reaction time in DES D.

**Scheme 2:**
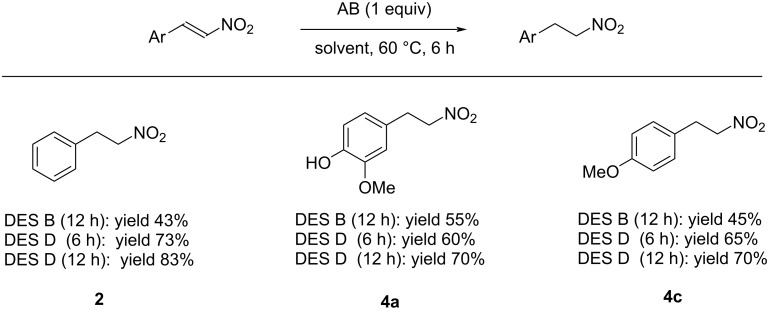
AB-mediated reductions of nitrostyrenes **1**, **3a**, and **3c** using DESs B and D.

Based on these findings, the reduction of some selected substrates in betaine/glycolic acid mixtures was reinvestigated (Scheme 2). We were pleased to see that after only 6 h of reaction in DES D, the corresponding nitroalkanes were obtained in good to excellent yield as very clean products. Notably, the formation of byproducts was not detected, even in traces. It is also worth mentioning that in DES D, it was possible to perform the reaction at a 0.75 M concentration, while with other deep eutectic mixtures and in glycerol, it was necessary to run the reaction in more diluted solutions (0.1 M) to obtain a better yield.

**Scheme 3:**
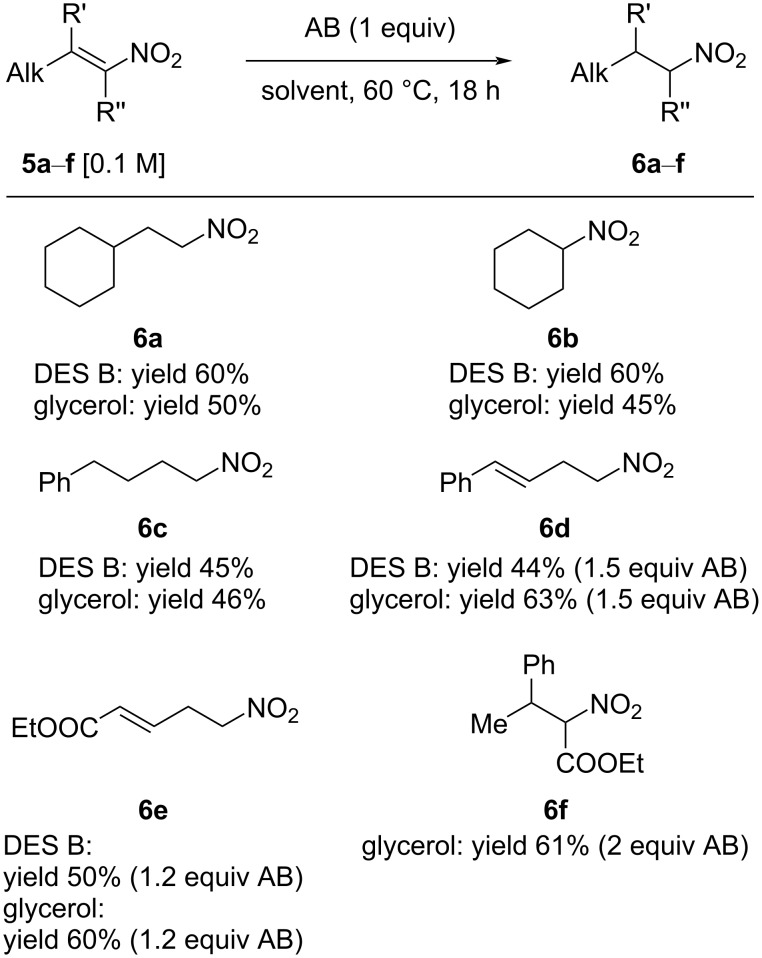
AB-mediated reductions of nitroalkenes **5a**–**f**.

Ammonia borane also proved to be an efficient reagent for the reduction of both linear and branched aliphatic nitroolefins, affording the expected products in fair to good yield (Scheme 3).

**Scheme 4:**
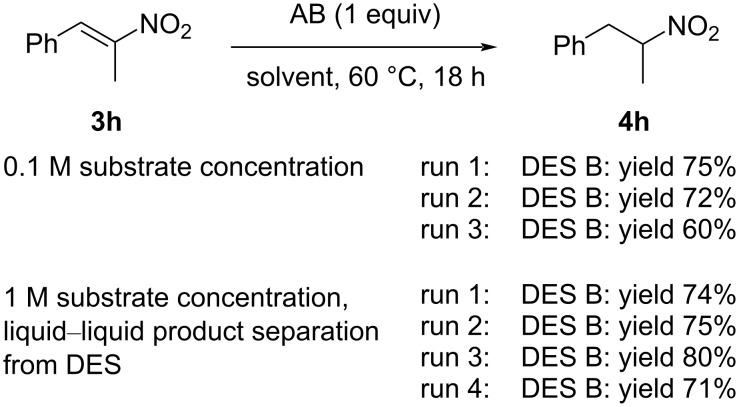
Recovery and recycling experiments in the AB-mediated reduction of nitrostyrene **3h** to afford nitroalkane **4h**.

The reduction of (2-nitrovinyl)cyclohexane (**5a**) afforded product **6a** in 60% yield in DES B. Analogously, nitrocyclohexene was reduced in 60% yield in a ChCl/glycerol mixture, while **6c** was obtained in similar yields in glycerol or DES B. Noteworthy, the reduction of nitrobutadiene **5d** produced nitro derivative **6d** in 63% yield in glycerol and 44% in DES B, with complete control of the chemoselectivity. Remarkably, the reduction of nitrodiene **5e**, featuring an ester group, was also accomplished with complete chemoselectivity in 50% yield in DES B and in 60% yield in glycerol. The reduction of the nitro group to an amine, followed by cyclization would afford the unsaturated δ-lactam, a valuable precursor for further synthetic elaborations. Noteworthy, AB was also able to efficiently reduce both isomers of tetrasubstituted nitroacrylate **5d**, which showed poor reactivity in the Hantzsch ester-mediated reduction in toluene, where only a low yield of the product could be obtained. However, in glycerol, the reaction of **5d** with ammonia borane afforded product **6d** in 61% isolated yield as 1:1 mixture of diastereomers (the diastereomeric ratio was not influenced by the reaction time, temperature or concentration). Finally, the possibility to develop a reliable, convenient protocol for the isolation of the nitroalkane and the recycling of the DES mixture was investigated (Scheme 4).

The first studies moved from the standard work-up procedure for the reaction performed at 0.1 M substrate concentration, which typically involves the addition of water to dissolve the DES and a small quantity of ethyl acetate to favor the extraction of the product and its separation from the eutectic mixture. However, when the recycling protocol was studied, in order to reuse the DES, at the end of the reaction, water was not added, and the product was extracted by simply adding ethyl acetate and separating the upper, organic phase. The DES phase was then reused as such, without any purification. After the first reaction (75% yield), DES B was reused in a second run that afforded the product in 72% yield; however, in the third run, a decreased yield was observed.

In order to realize a more convenient and more efficient protocol that avoids the use of any organic solvent, the reduction of **3h** was performed with a 1 M substrate concentration and afforded, in DES B, nitroalkane **4h** in 74% yield, which was isolated as pure compound by a simple liquid–liquid biphase separation from the eutectic mixture (see details and pictures in Supporting Information File 1). The DES was reused other three times with no appreciable difference in the yield.

## Conclusion

In conclusion, the combination of an atom economically convenient and inexpensive reagent such as ammonia borane with bio-based and biorenewable alternative solvents such as deep eutectic mixtures was efficiently used in the chemoselective reduction of nitroalkenes to afford the corresponding nitroalkanes in fair to good yield. The possibility to isolate the product without the addition of any organic solvent and to recycle the eutectic mixtures at least three times in further reactions was also demonstrated. A wide variety of alkyl- and aryl-substituted nitroalkenes were reduced with high chemoselectivity, including β-substituted nitroolefins, and thus paving the way to the study of innovative, sustainable and stereoselective reductive methods to synthesize enantiopure nitroalkanes, valuable precursors of chiral amines.

## Experimental

### Experimental procedure for the reduction in glycerol

The desired nitroolefin (0.4 mmol) was suspended in glycerol (4 mL) in a 7 mL vial equipped with a 3.5 cm-long magnetic stir bar. Ammonia borane (12 mg, 0.4 mmol) was added to the suspension at room temperature, and the reaction flask was placed in an oil bath (already heated at 60 °C). After 18 h, the reaction mixture was cooled to room temperature, and it was diluted with 4 mL of water. The product was extracted with ethyl acetate (3 × 4 mL). The combined organic phases were washed with water (2 × 4 mL), dried over Na_2_SO_4_, filtered, and the solvent was evaporated under reduced pressure. The crude product was purified by column chromatography (silica as stationary phase; eluent: *n*-hexane/ethyl acetate).

### Experimental procedure for the reduction in DES

In a 7 mL vial with a 3.5 cm-long magnetic stir bar, 4.5 g of DES were freshly prepared, ChCl and glycerol (1:2 molar ratio) were mixed, and the mixture was heated at 70 °C for 15 min until it became a colorless liquid. Then, the DES was slowly cooled to room temperature in 15 min. The desired nitroolefin (0.4 mmol) was suspended in the DES, ammonia borane (12 mg, 0.4 mmol) was added to the suspension, and the reaction mixture was heated at 60 °C. After 18 h, the reaction mixture was cooled to room temperature, the DES was dissolved with the addition of 4 mL of water, and the product was extracted with AcOEt (3 × 4 mL). The combined organic phase was washed with water (2 × 4 mL), dried over Na_2_SO_4_, filtered, and the solvent was evaporated under reduced pressure. The crude product was purified by column chromatography (silica as stationary phase; eluent: *n*-hexane/ethyl acetate).

### Recycling experiments with liquid–liquid biphasic separation

In a 7 mL vial with a 3.5 cm-long magnetic stir bar, 2.5 g of DES were freshly prepared, ChCl and glycerol (1:2 molar ratio) were mixed, and the mixture was heated at 70 °C for 15 min until it became a colorless liquid. Then, the DES was slowly cooled to room temperature in 15 min. *trans*-β-Methyl-β-nitrostyrene (326 mg, 2 mmol) was suspended in the DES. Ammonia borane (62 mg, 2 mmol) was added to the suspension, and the reaction mixture was heated at 60 °C. After 18 h, the reaction mixture was cooled to room temperature, centrifuged, and the product was removed by liquid–liquid separation with a Pasteur pipette. The crude product was purified by column chromatography (silica as stationary phase; eluent: *n*-hexane/ethyl acetate).

For the subsequent runs, fresh β-methyl-β-nitrostyrene (326 mg, 2 mmol) was suspended in the DES, and ammonia borane (1 equiv) was added to the mixture. The procedure is analogous to the first run.

## Supporting Information

File 1 Experimental setup and general procedures for the reduction reactions as well as product characterization and NMR data.
