# Supplementary material for: Nitroalkene reduction in deep eutectic solvents promoted by BH3NH3
Source: Beilstein J Org Chem. 2021 May 6;17:1041–7. doi: 10.3762/bjoc.17.83 (PMC8111428; doi:10.3762/bjoc.17.83)

BEILSTEIN JOURNAL OF ORGANIC CHEMISTRY

Supporting Information

for

Nitroalkene reduction in deep eutectic solvents promoted by BH_3NH_3

Chiara Faverio, Monica Fiorenza Boselli, Patricia Camarero Gonzalez, Alessandra Puglisi and Maurizio Benaglia

Beilstein J. Org. Chem. **2021,** *17,* 1041–1047. doi:10.3762/bjoc.17.83

Experimental setup and general procedures for the reduction reactions as well as product characterization and NMR data

License and Terms: This is a supporting information file under the terms of the Creative Commons Attribution License (https://creativecommons.org/licenses/by/4.0). Please note that the reuse, redistribution and reproduction in particular requires that the author(s) and source are credited and that individual graphics may be subject to special legal provisions.
The license is subject to the *Beilstein Journal of Organic Chemistry* terms and conditions: (https://www.beilstein-journals.org/bjoc/terms)

Table of Contents:

General methods and materials	S2
Synthesis of nitroalkenes	S3
General procedure for nitroalkenes reduction	S4
Products characterization	S7
Selected NMR spectra	S12

General methods and materials

Commercial grade reagents and solvents were used without further purifications. *trans*-β-Nitrostyrene **1** (CAS 5153-67-3), *trans*-β-Methyl-β-nitrostyrene **3h** (CAS 705-60-2) and 1-Nitro-1-cyclohexene **5b** (CAS 2562-37-0) were purchased by Sigma Aldrich and they were used without further purifications. The synthesis of the nitroolefines **3a-g**, **5a**, **5c**, **5d** is reported in a previous publication (C. Faverio, M. F. Boselli, L. Raimondi, M. Benaglia, *SynOpen* **2020**, *4*, 116.)

Ammonia borane (CAS 13774-81-7) was purchased by Sigma Aldrich and it was used without further purifications.

NMR spectra: ^{1}H-NMR and ^{13}C-NMR spectra were recorded with instruments at 300 MHz (Bruker AV 300). The chemical shifts are reported in ppm (δ), with the solvent reference relative to tetramethylsilane (TMS).

Mass spectra: Mass spectra and accurate mass analysis were carried out on a VG AUTOSPEC-M246 spectrometer (double-focusing magnetic sector instrument with EBE geometry) equipped with LCQ Fleet ion trap mass spectrometer, ESI source, with acquisition in positive ionization mode in the mass range of 50-2000 m/z.

TLC: Reactions and chromatographic purifications were monitored by analytical thin-layer chromatography (TLC) using silica gel 60 F254 pre-coated glass plates and visualized using UV light, vanillin or $KMnO_4$.

Chromatographic purification: Purification of the products was performed by column chromatography with flash technique (according to the Still method) using as stationary phase silica gel 230-400 mesh (SIGMA ALDRICH).

Reaction with microwave apparatus: the reactions were conducted with CEM Discover SP microwave; with an irradiation power of 200 W and reaction temperature of 90 °C for 1 hour.

Caution: Work in a well-ventilated space and avoid contact with easily reduced, flammable compounds (e.g. acetone), which may combust upon contact with ammonia borane.

Synthesis of nitroalkenes

Synthesis of (2E,4E)-ethyl 5-nitropenta-2,4-dienoate (**5e**)

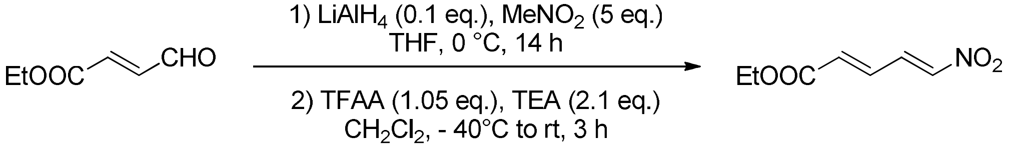

The compound was prepared from ethyl *trans*-4-oxo-2-butenoate and nitromethane according to a published procedure.[1] The title product was isolated after column chromatography as a pale yellow solid. All analytical data are in agreement with the literature.

^{1}H-NMR (300 MHz, $CDCl_3$): δ 7.61 (t, J = 12.5 Hz, 1H), 7.35-7.27 (m, 2H), 6.43 (d, J = 15.5 Hz, 1H), 4.27 (q, J = 7.1 Hz, 2H), 1.33 (t, J = 7.1 Hz, 3H) ppm.

Synthesis of methyl 2-nitro-3-phenylbut-2-enoate (**5f**)

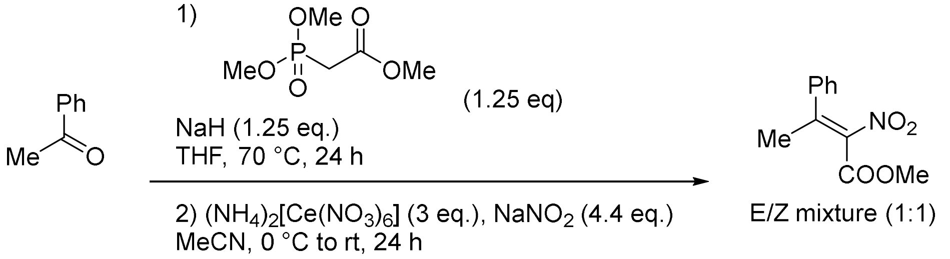

The compound was prepared from acetophenone according to a published procedure.[2] The title product was isolated after column chromatography as a yellow oil.

^{1}H NMR (300 MHz, $CDCl_3$): δ 7.44 – 7.38 (m, 6H isomer A+B), 7.30 – 7.24 (m, 4H isomer A+B), 3.88 (s, 3H isomer A), 3.63 (s, 3H isomer B), 2.58 (s, 3H isomer A), 2.35 (s, 3H isomer B) ppm.
^{13}C NMR (75 MHz, $CDCl_3$): δ 164.22, 160.04, 159.95, 150.25, 149.35, 141.93, 141.54, 137.74, 137.29, 129.63, 129.57, 128.87, 128.67, 126.69, 125.99, 122.68, 52.96, 52.89, 22.92, 21.58 ppm.
HRMS (ESI +) m/z calculated for $C_{11}H_{11}NO_4Na$ [M+Na]: 244.0586, found: 244.0589.

[1] S. Belota, A. Quintarda, N. Krauseb, A. Alexakis, *Adv. Synth. Catal* **2010**, *352*, 667.

[2] First step: J. Wen, J. Jian, X. Zhang, *Org. Lett*. **2016**, *18*, 4451.
Second step: A. V. Buevich, Y. Wu, T. Chan, A. Stamford, *Tetrahedron Letters*, **2008**, *49*, 2132.

General procedure A: nitroalkenes reduction in DES B

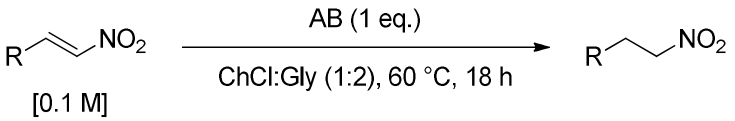

In a 7 mL vial with a 3.3 cm long magnetic stir bar, 4.5 g of DES were freshly prepared, choline chloride and glycerol (1:2 molar ratio) were mixed and the mixture was heated at 70 °C for 15 minutes until it became a colourless liquid. Then, the DES was slowly cooled down to room temperature in 15 minutes. The desired nitroolefin (0.4 mmol) was suspended into the DES, ammonia borane (12 mg, 0.4 mmol) was added to the suspension, and the reaction mixture was heated at 60°C. After 18 hours, the reaction mixture was cooled to room temperature, the DES was dissolved with the addition of 4 mL of water and the product was extracted with AcOEt (4 mL x 3). The combined organic phase was washed with water (4 mL x 2), dried over Na_2SO_4, filtered, and the solvent was evaporated under reduced pressure. The crude product was purified by column chromatography (Silica as stationary phase; eluent *n*-hexane/ethyl acetate).

General procedure B: nitroalkenes reduction in glycerol

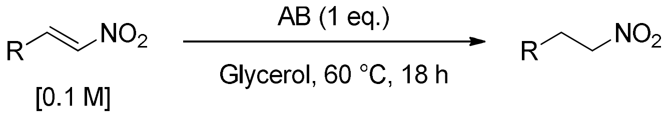

The desired nitroolefin (0.4 mmol) was suspended in glycerol (4 mL) in a 7 mL vial equipped with 3.3 cm a long magnetic stir bar. Ammonia borane (12 mg, 0.4 mmol) was added to the suspension at room temperature and the reaction flask was placed in an oil bath (already heated at 60 °C). After 18 hours, the reaction mixture was cooled to room temperature and it was diluted with 4 mL of water. The product was extracted with ethyl acetate (4 mL x 3). The combined organic phases were washed with water (4 mL x 2), dried over Na_2SO_4, filtered and the solvent was evaporated under reduced pressure. The crude product was purified by column chromatography (Silica as stationary phase; eluent *n*-hexane/ethyl acetate).

General procedure C: nitroalkenes reduction in DES D

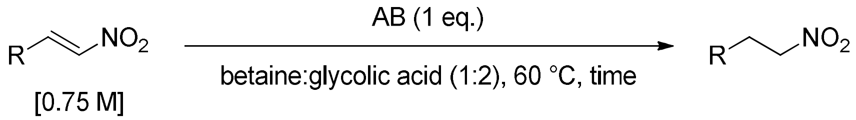

In a 3 mL vial with a 1.6 cm long magnetic stir bar, 667 mg of DES were freshly prepared, anhydrous betaine and glycolic acid (1:2 molar ratio) were mixed and the mixture was heated at 85 °C for 1 hour until it became a colourless liquid. Then, the DES was slowly cooled down to room temperature in 15 minutes. The desired nitroolefin (0.4 mmol) was suspended into the DES, ammonia borane (12 mg, 0.4 mmol) was added to the suspension, and the reaction mixture was heated at 60°C. After the desired time, the reaction mixture was cooled to room temperature, the DES was dissolved with the addition of 2 mL of water and the product was extracted with AcOEt (3 mL x 3). The combined organic phase was washed with water (3 mL x 2), dried over Na_2SO_4, filtered, and the solvent was evaporated under reduced pressure. The crude product was purified by column chromatography (Silica as stationary phase; eluent *n*-hexane/ethyl acetate).

Recycling experiments with liquid-liquid biphasic separation of the product

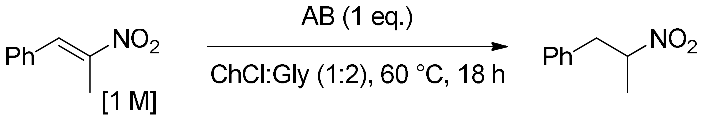

In a 7 mL vial with a 3.3 cm long magnetic stir bar, 2.5 g of DES were freshly prepared, choline chloride and glycerol (1:2 molar ratio) were mixed and the mixture was heated at 70 °C for 15 minutes until it became a colourless liquid. Then, the DES was slowly cooled down to room temperature in 15 minutes. *trans*-β-Methyl-β-nitrostyrene (326 mg, 2 mmol) was suspended into the DES. Ammonia borane (62 mg, 2 mmol) was added to the suspension, and the reaction mixture was heated at 60°C. After 18 hours, the reaction mixture was cooled to room temperature, it was centrifuged and the product was removed by liquid-liquid separation with a Pasteur pipette. The crude product was purified by column chromatography (Silica as stationary phase; eluent *n*-hexane/ethyl acetate).

For the subsequent runs, fresh methyl-β-nitrostyrene (326 mg, 2 mmol) was suspended into the DES and ammonia borane (1 eq.) was added to the mixture. The procedure is analogue to the first run.

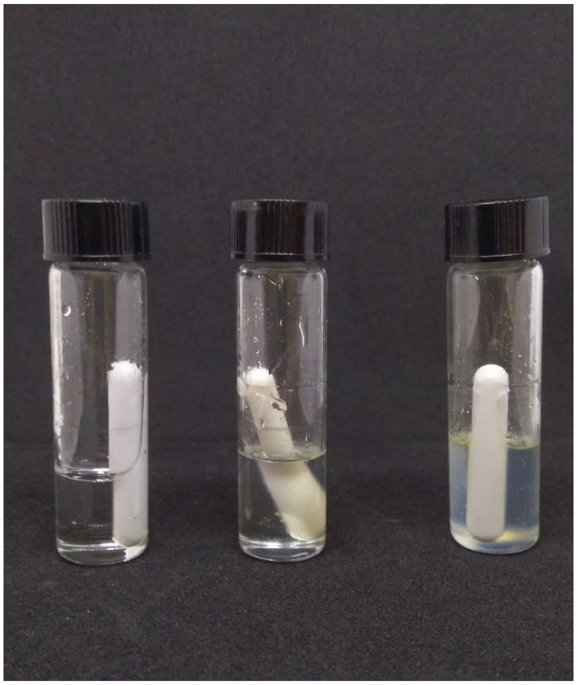

Figure 1 – From left, a vial with 2.5g of freshly prepared DES, a vial with DES used in two runs and a vial with DES used in four runs

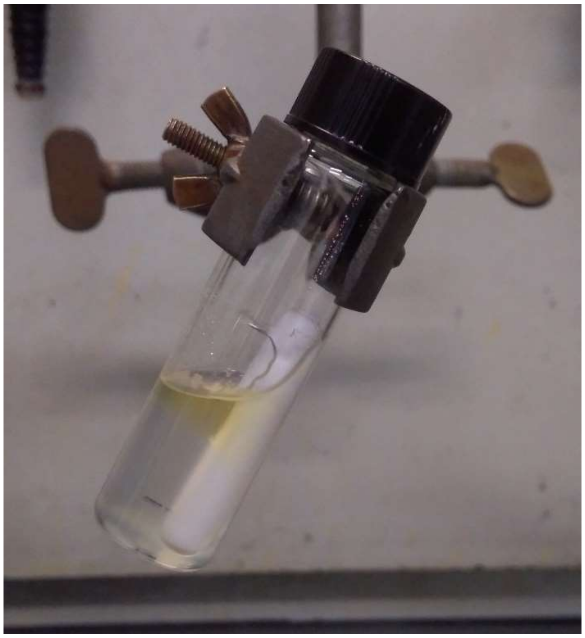

Figure 2 – liquid-liquid separation of the product (pale yellow oil) from the DES

Products characterization

2-Phenylnitroethane (**2**)

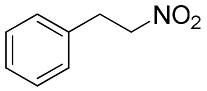

Prepared according to general procedure A, B or C. The crude mixture was purified by column chromatography on silica gel eluting with hexane/ethyl acetate 95:5 afford the title product as a colorless oil. All analytical data are in agreement with literature.[3]

2-methoxy-4-(2-nitroethyl)phenol (**4a**)

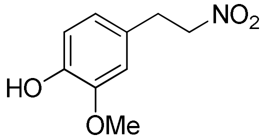

Prepared according to general procedure A, B or C. The crude mixture was purified by column chromatography on silica gel eluting with hexane/ethyl acetate 65:35 afford the title product as a bright yellow oil (55% yield with procedure A, 70% yield with procedure B, 70% yield with procedure C). All analytical data are in agreement with literature.[4]

1,3-dimethoxy-2-(2-nitroethyl)benzene (**4b**)

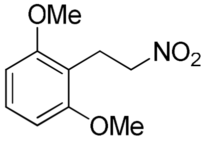

Prepared according to general procedure A or B. The crude mixture was purified by column chromatography on silica gel eluting with hexane/ethyl acetate 8:2 afford the title product as a pale-yellow oil (75% yield with procedure A, 65% yield with procedure B). All analytical data are in agreement with literature. [5]

1-methoxy-4-(2-nitroethyl)-benzene (**4c**)

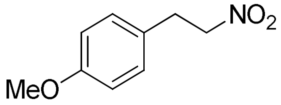

Prepared according to general procedure A, B or C. The crude mixture was purified by column chromatography on silica gel eluting with hexane/ethyl acetate 8:2 afford the title product as a bright yellow oil (45% yield with procedure A, 80% yield with procedure B, 70% yield with procedure C). All analytical data are in agreement with literature.[6]

2-*p*-tolylnitroethane (**4d**)

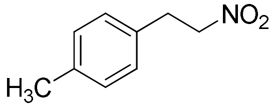

Prepared according to general procedure A or B. The crude mixture was purified by column chromatography on silica gel eluting with hexane/ethyl acetate 95:5 afford the title product as a pale-yellow oil (43% yield with procedure A, 70% yield with procedure B). All analytical data are in agreement with literature.[7]

---

[3] P. Vojáčková, D. Chalupa, J. Prieboj, M. Nečas, J. Švenda, *Org. Lett.* **2018**, *20*, 7085.
[4] J. Wangand, G. Evano, *Org. Lett.* **2016**, *18*, 3542.
[5] C. Faverio, M. F. Boselli, L. Raimondi, M. Benaglia, *SynOpen* **2020**, *4*, 116.
[6] S. Rezazadeh, V. Devannah, D. A. Watson, *J. Am. Chem. Soc.*, **2017**, *139*, 8110.
[7] M. Marčeková, P. Gerža, M. Šoral, J. Moncol, D. Berkeš, A. Kolarovič, P. Jakubec, *Org. Lett.*, **2019**, *21*, 4580.

1-(4-chlorophenyl)-2-nitroethane (**4e**)

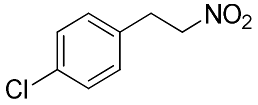

Prepared according to general procedure A or B (two equivalents of ammonia borane were used). The crude mixture was purified by column chromatography on silica gel eluting with hexane/ethyl acetate 95:5 afford the title product as a yellow oil (32% yield with procedure A, 66% yield with procedure B). All analytical data are in agreement with literature.[8]

1-(4-bromophenyl)-2-nitroethane (**4f**)

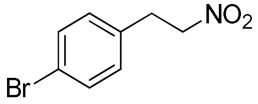

Prepared according to general procedure A or B (two equivalents of ammonia borane were used). The crude mixture was purified by column chromatography on silica gel eluting with hexane/ethyl acetate 95:5 afford the title product as a pale-yellow oil (41% yield with procedure A, 73% yield with procedure B). All analytical data are in agreement with literature.[9]

[8] Y. M. McNamaraa, S. M. Cloonanb, A. J. S. Knoxb, J. J. Keatinga, S. G. Butlera, G. H. Petersc, M. J. Meegana, D. C. Williams, *Bioorg. Med. Chem*, **2011**, *19*, 1328.
[9] S. Mahesh, V. Adebomi, Z. P. Muneeswaran, M. Raj, *Angew.Chem. Int.Ed.*, **2020**, *59*,2793.

1-nitro-2-phenylpropane (**4g**)

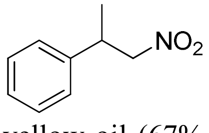

Prepared according to general procedure A or B (two equivalents of ammonia borane were used). The crude mixture was purified by column chromatography on silica gel eluting with hexane/ethyl acetate 95:5 afford the title product as a yellow oil (67% yield with procedure A, 74% yield with procedure B). All analytical data are in agreement with literature.[10]

2-nitro-1-phenyl-propane (**4h**)

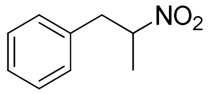

Prepared according to general procedure A or B. The crude mixture was purified by column chromatography on silica gel eluting with hexane/ethyl acetate 95:5 afford the title product as a pale-yellow oil (75% yield with procedure A, 63% yield with procedure B). All analytical data are in agreement with literature.[11]

2-cyclohexyl-1-nitroethane (**6a**)

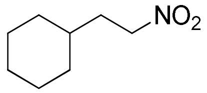

Prepared according to general procedure A or B. The crude mixture was purified by column chromatography on silica gel eluting with hexane/ethyl acetate 95:5 afford the title product as a pale-yellow oil (60% yield with procedure A, 50% yield with procedure B). All analytical data are in agreement with literature.[12]
Caution! Efficient mixing and inert atmosphere are required to perform the reaction, while avoiding the uncontrolled reaction between the substrate and the reducing agent.

Nitrocyclohexane (**6b**)

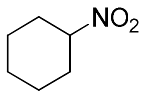

Prepared according to the general procedure A or B. The crude mixture was purified by column chromatography on silica gel eluting with *n*-pentane/diethyl ether 99:1 to afford the title product as a colourless oil (60% yield with procedure A, 45% yield with procedure B). All analytical data are in agreement with literature.[12]
Caution! Efficient mixing and inert atmosphere are required to perform the reaction, while avoiding the uncontrolled reaction between the substrate and the reducing agent.

1-nitro-4-phenylbutane (**6c**)

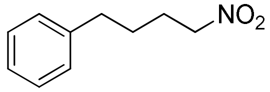

Prepared according to general procedure A or B. The crude mixture was purified by column chromatography on silica gel eluting with hexane/ethyl acetate 9:1 afford the title product as a colourless oil (45% yield with procedure A, 46% yield with procedure B). All analytical data are in agreement with literature.[13]

---

[10] T. Hostmann, J. J. Molloy, K. Bussmann, R. Gilmour, *Org. Lett.*, **2019**, *21*, 10164.
[11] J. Xianga, E. Sunb, C. Liana, W. Yuana, J. Zhua, Q. Wanga, J. Deng, *Tetrahedron*, **2012**, *68*, 4609.
[12] Z. Zhang, P. R. Schreiner, *Synthesis*, **2007**, *16*, 2559.

(*E*)-(4-nitrobut-1-en-1-yl)benzene (**6d**)

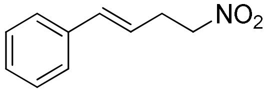

Prepared according to general procedure A or B (1.5 equivalents of ammonia borane were used). The crude mixture was purified by column chromatography on silica gel eluting with hexane/ethyl acetate 95:5 afford the title product as a yellow oil (44% yield with procedure A, 63% yield with procedure B). All analytical data are in agreement with literature.[14]

(*E*)-ethyl 5-nitropent-2-enoate (**6e**)

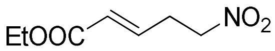

Prepared according to the general procedure A or B. The crude mixture was purified by column chromatography on silica gel eluting with hexane/ethyl acetate 65:35 to afford the title product as a colourless oil (50% yield with procedure A, 60% yield with procedure B).

^{1}H-NMR (300 MHz, $CDCl_3$): δ 6.85 (dt, J = 15.7, 6.9 Hz, 1H), 5.94 (dt, J = 15.7, 1.5 Hz, 1H), 4.50 (t, J = 6.9 Hz, 2H), 4.19 (q, J = 7.1 Hz, 2H), 2.90 (qd, J = 6.9, 1.5 Hz, 2H), 1.28 (t, J = 7.1 Hz, 3H) ppm. **^{13}C NMR** (75 MHz, $CDCl_3$): δ 165.70, 141.06, 125.13, 73.44, 60.76, 29.52, 14.32 ppm. **HRMS (ESI +)** m/z calculated for $C_7H_{11}NO_4Na$ [M+Na]: 196.0586, found: 196.0582.

---

[13] R. Matsubara, H. Kim, T. Sakaguchi, W. Xie, X. Zhao, Y. Nagoshi, C. Wang, M. Tateiwa, A. Ando, M. Hayashi, M. Yamanaka, T. Tsuneda, *Org. Lett.*, **2020**, *22*, 1182.

[14] R. Padilla–Salinas, R. R. Walvoord, S. Tcyrulnikov, M. C. Kozlowski, *Org. Lett.*, **2013**, *15*, 3966.

Methyl 2-nitro-3-phenylbutanoate (**6f**)

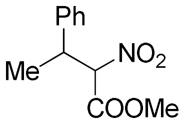

1:1 mixture of diastereoisomers

Prepared according to general procedure B (2 equivalents of ammonia borane were used). The crude mixture was purified by column chromatography on silica gel eluting with hexane/ethyl acetate 95:5 afford the title product as a colourless oil (61% yield).

^{1}H-NMR (300 MHz, $CDCl_3$): δ 7.39 – 7.24 (m, 10H diast A+B), 5.30 (dd, J = 13.0, 10.2 Hz, 2H diast A+B), 3.89 (s, 3H diast A), 3.87 – 3.77 (m, 2H diast A+B), 3.59 (s, 3H diast B), 1.45 (d, J = 6.9 Hz, 3H diast A), 1.41 (d, J = 7.1 Hz, 3H diast B). **^{13}C NMR** (75 MHz, $CDCl_3$): δ 164.24, 163.84, 139.96, 139.04, 129.07, 128.05, 128.00, 127.82, 127.40, 93.49, 93.22, 53.72, 53.40, 41.45, 41.31, 18.30. **HRMS (ESI +)** m/z calculated for $C_{11}H_{13}NO_4Na$ [M+Na]: 246.0742, found: 246.0742.

Selected NMR spectra

(*E*)-ethyl 5-nitropent-2-enoate (**6e**) – ^{1}H NMR

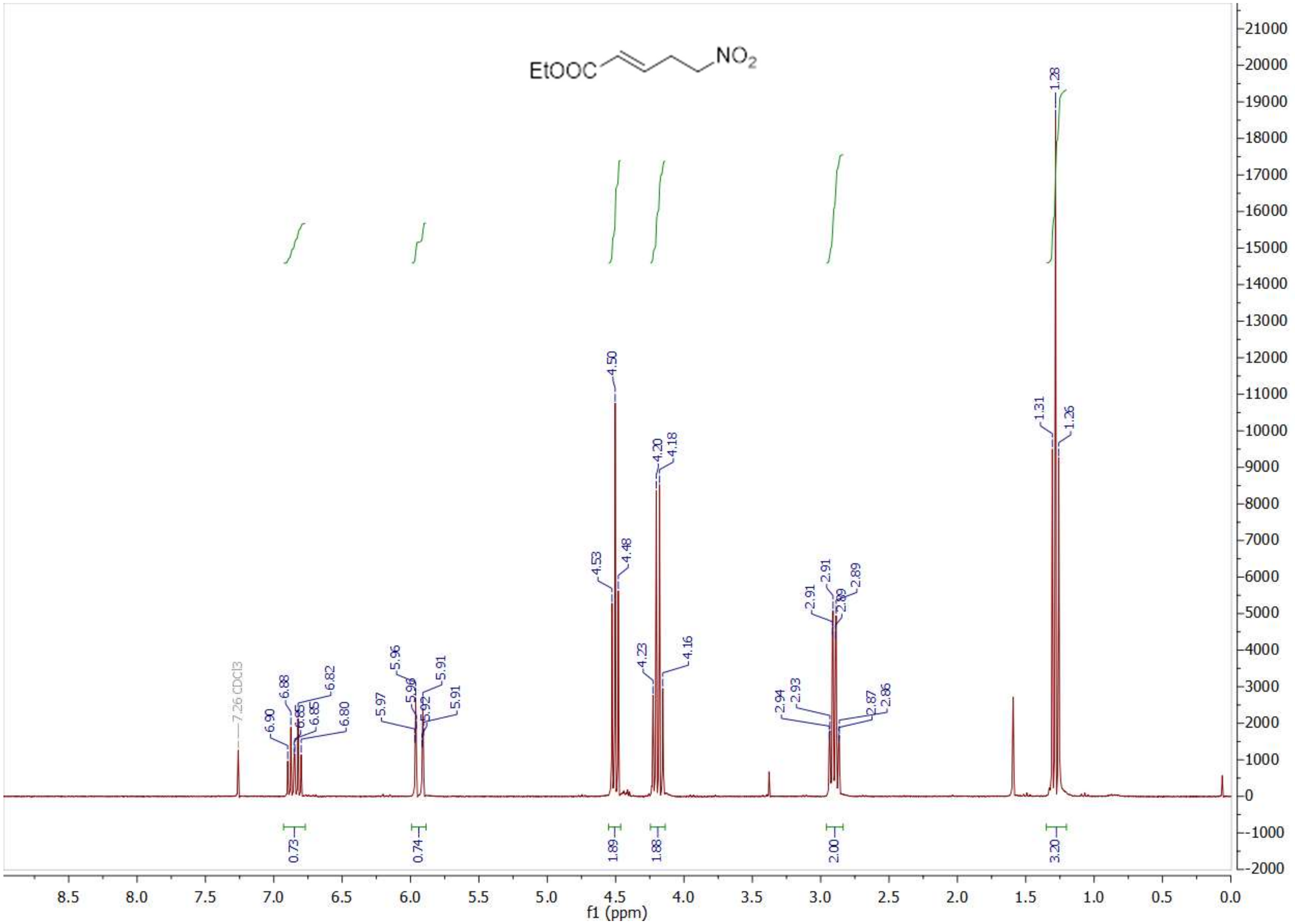

(*E*)-ethyl 5-nitropent-2-enoate (**6e**) – ^{13}C NMR

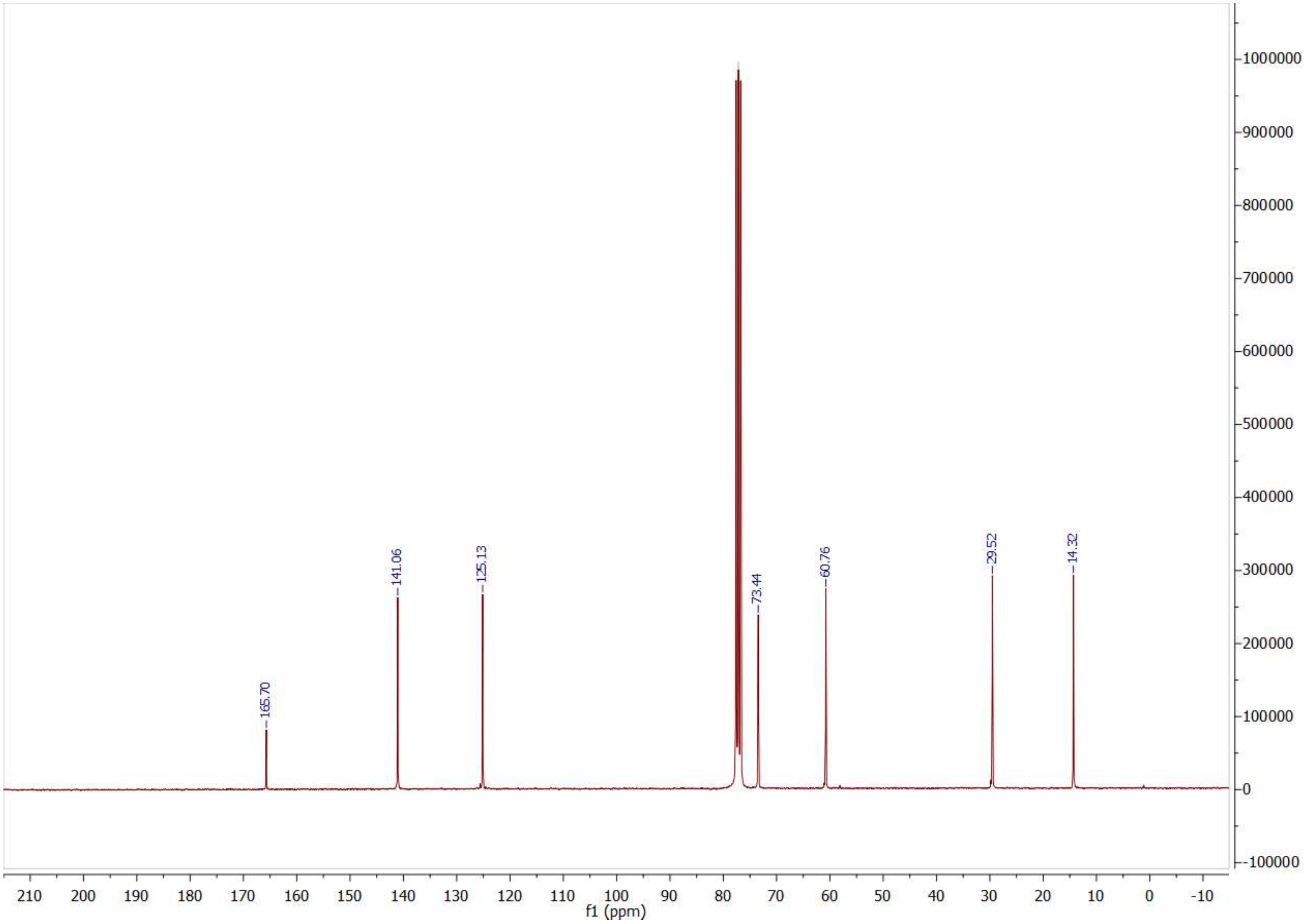

Methyl 2-nitro-3-phenylbutanoate (**6f**) – ^{1}H NMR

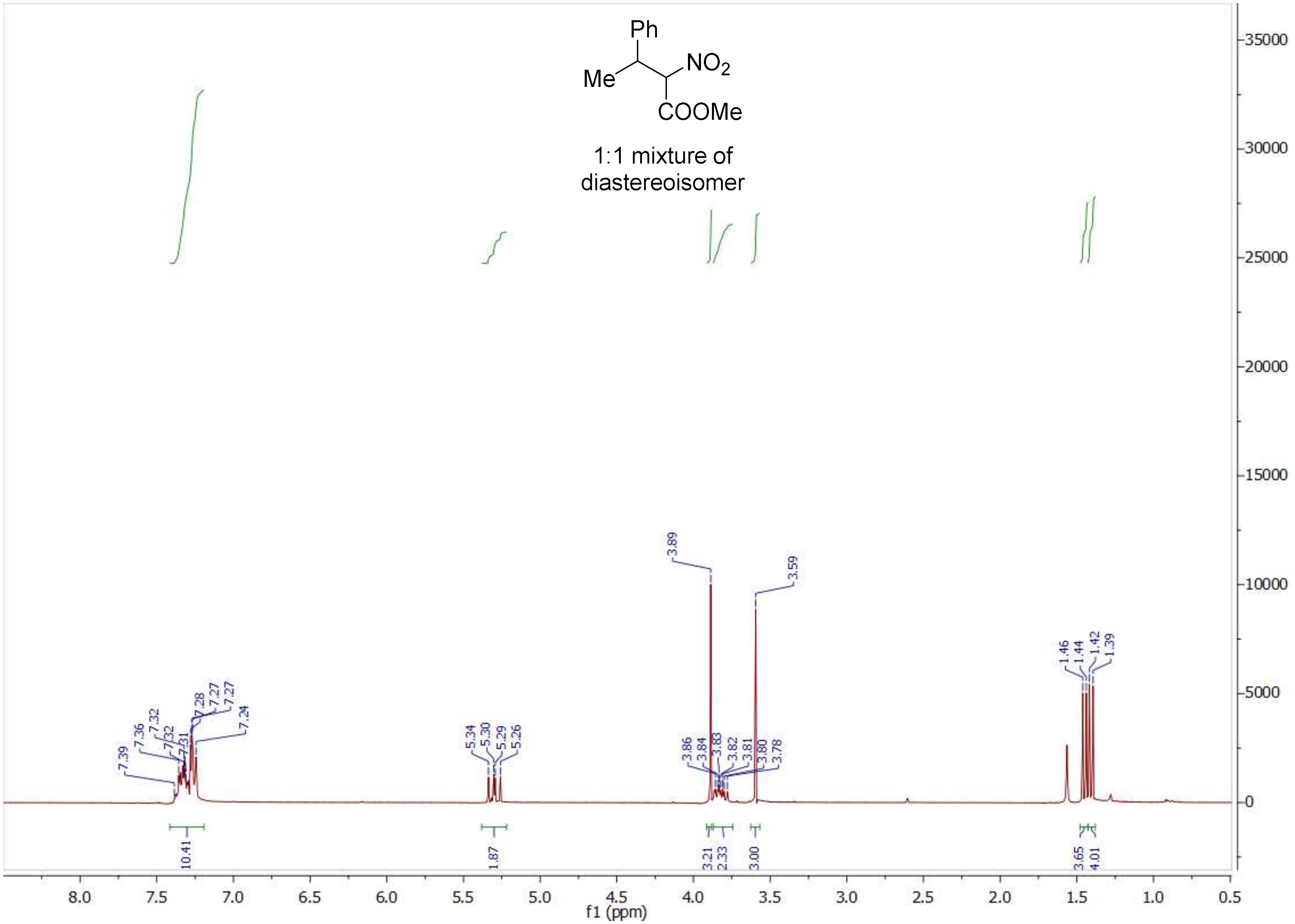

^{1}H NMR of the crude mixture (liquid/liquid separation of the product) – recycling test run #2

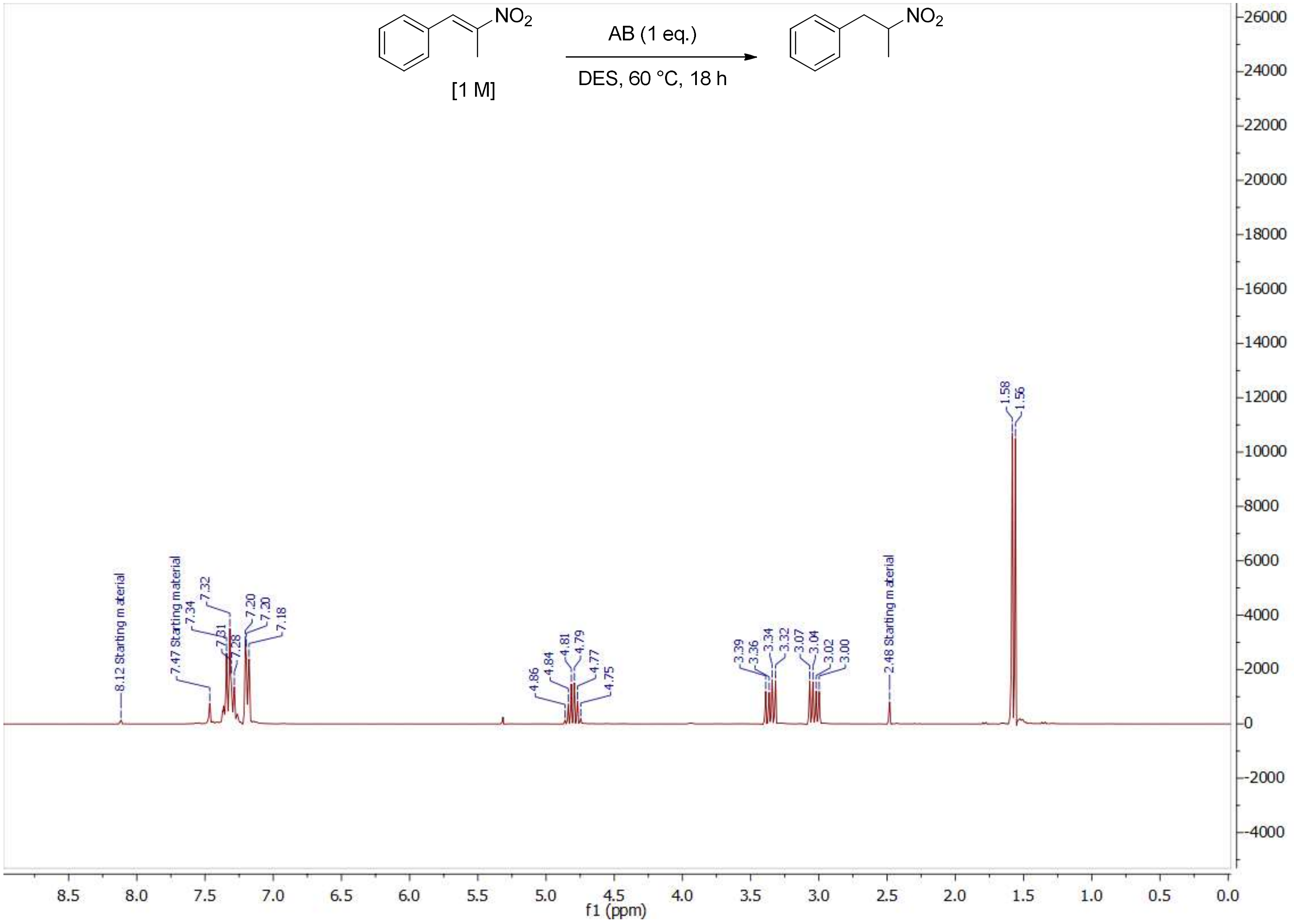

^{1}H NMR of the purified product - 2-nitro-1-phenyl-propane (**4g**) – recycling test run #2

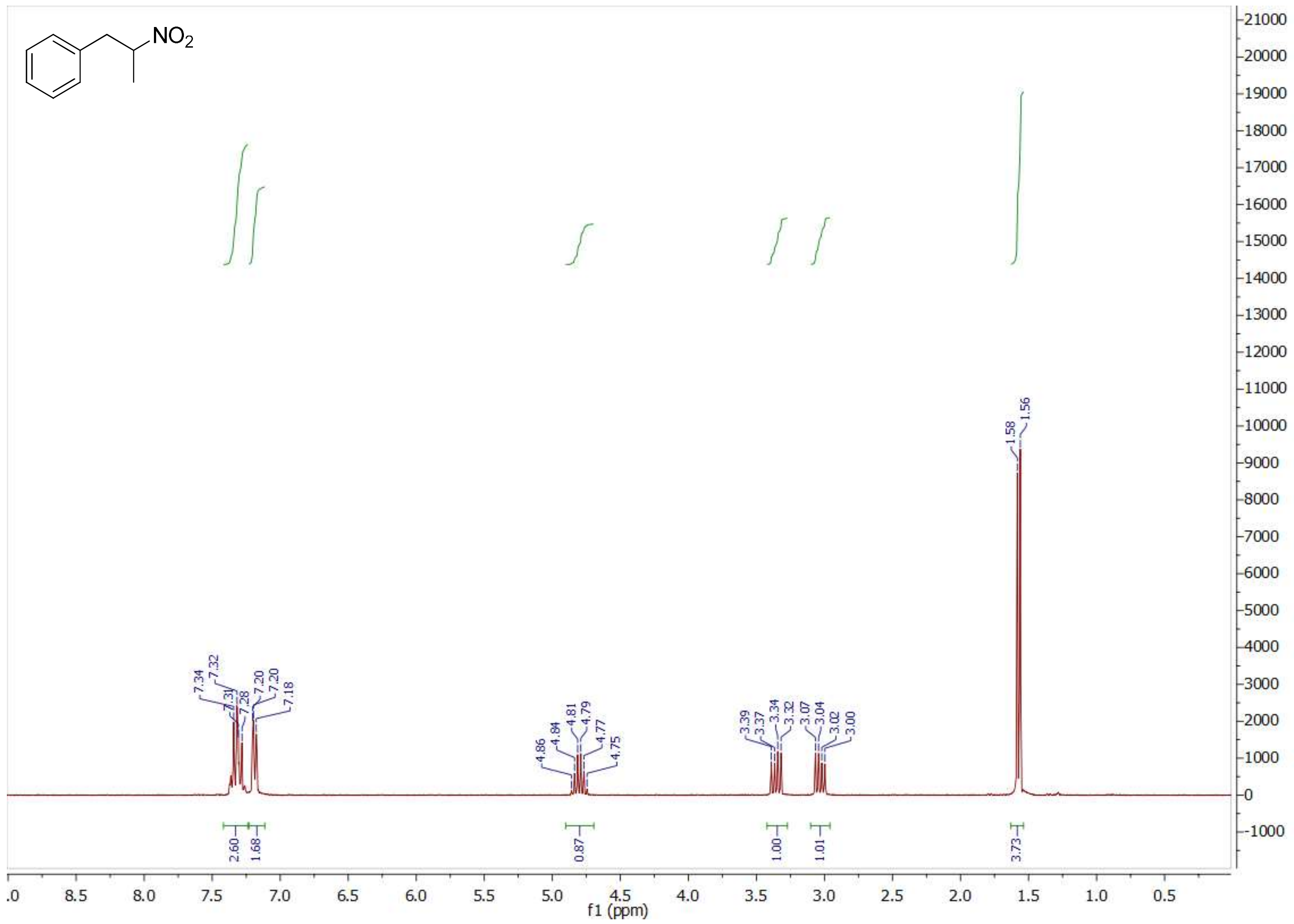

Supplement: File 1 — Experimental setup and general procedures for the reduction reactions as well as product characterization and NMR data. [file Beilstein_J_Org_Chem-17-1041-s001.pdf]
